# Osteocyte Apoptosis Contributes to Cold Exposure-induced Bone Loss

**DOI:** 10.3389/fbioe.2021.733582

**Published:** 2021-11-11

**Authors:** Jingke Du, Zihao He, Junqi Cui, Hanjun Li, Mingming Xu, Shuhong Zhang, Shuangyan Zhang, Mengning Yan, Xinhua Qu, Zhifeng Yu

**Affiliations:** ^1^ Shanghai Key Laboratory of Orthopedic Implants, Department of Orthopedic Surgery, Shanghai Ninth People’s Hospital, Shanghai Jiao Tong University School of Medicine, Shanghai, China; ^2^ Arthritis Clinic and Research Center, Peking University People’s Hospital, Peking University, Beijing, China; ^3^ Department of Pathology, Shanghai Ninth People’s Hospital, Shanghai Jiao Tong University School of Medicine, Shanghai, China; ^4^ Department of Bone and Joint Surgery, Renji Hospital, School of Medicine, Shanghai Jiao Tong University, Shanghai, China

**Keywords:** cold exposure, bone mass, bone remodeling, osteocyte, perilacunar /canalicular remodeling

## Abstract

Emerging evidence indicates that bone mass is regulated by systemic energy balance. Temperature variations have profound effects on energy metabolism in animals, which will affect bone remodeling. But the mechanism remains unclear. 2-month-old C57BL/6J male mice were exposed to cold (4°C) and normal (23°C) temperatures for 28 days and the effects of cold exposure on bone mass was investigated. Micro-computed tomography results showed that bone volume fraction was significantly reduced after 14 days of exposure to cold temperature, and it was recovered after 28 days. Ploton silver staining and immunohistochemical results further revealed that exposure to cold decreased canalicular length, number of E11-and MMP13-positive osteocytes after 14 days, but they returned to the baseline levels after 28 days, different from the normal temperature control group. In addition, change of Caspase-3 indicated that exposure to cold temperature augmented apoptosis of osteocytes. *In vitro* results confirmed the positive effect of brown adipocytes on osteocyte‘s dendrites and E11 expression. In conclusion, our findings indicate that cold exposure can influence bone mass in a time-dependent manner, with bone mass decreasing and recovering at 2 and 4 weeks respectively. The change of bone mass may be caused by the apoptosis osteocytes. Brown adipocyte tissue could influence bone remodeling through affecting osteocyte.

## Introduction

Bone mass can be influenced by energy balance in many ways, and studies have demonstrated that caloric restriction and changes in leptin levels result in reduction of bone mass (Shi et al., 2008; Devlin et al., 2010). Likewise, the relationship between temperature and bone mass has aroused curiosity in recent years. It has been shown that low temperature has a negative influence on bone mass (Robbins et al., 2018), whereas warm temperature exposure (34°C) prevents ovariectomies from bone loss through the microbiota in the intestinal tract (Chevalier et al., 2020). Further studies have revealed that cold temperature can activate the sympathetic nerves, which promote non-shivering thermogenesis of the muscle and have a deleterious effect on bone mineral density (Bonnet et al., 2007; Chen et al., 2019). Wee et al. have shown that exposure to cold decreases bone mass in the neuropeptide Y (NPY) wild-type mice, whereas the absence of NPY in the null mice obliterates these changes in bone mineral density (Wee et al., 2020). Steinberg et al. have found that the bone diameter and cortical thickness in the femoral midshaft decreased significantly after 69 days but increased after 83 days of extreme cold (5°C) exposure (Steinberg et al., 1981). Thus, determining the relationship between long-term exposure to low temperatures and bone mass will further meaningful insights about the phenomenon. Additionally, identifying the decisive factors that play critical role in this phenomenon will be of crucial significance in developing effective therapeutic strategies for skeletal or bone disorders.

Osteocytes are the most abundant cell type in the bone. Morphologically, they are stellate cells with many dendrites around them that function as important mechanical sensors (Dallas et al., 2013; Iolascon et al., 2013; Choy et al., 2020). *In vivo*, dendrites of osteocytes are buried in the bone canaliculus, through which osteocytes can connect with each other to exchange biological signals, mechanosensations, and absorb nourishment from the interstitial fluid (Wang et al., 2004; Bonewald, 2011; Wang, 2018). Interstitial fluid pressures in the bone lacuna-canalicular system vary with loads applied to the bone and cause deformation of the osteocyte membrane (Weinbaum et al., 1994). Therefore, the integrity of the canaliculus is important for the bone to maintain a standard mechanical response. It has been reported that osteocytes are associated with unload-induced bone loss in BCL transgenic mice (Moriishi et al., 2012; Komori, 2013). Osteocytes can cause resorption or deposition of bone matrix through perilacunar/canalicular remodeling (Dole et al., 2020; Kegelman et al., 2020), which is another way for osteocytes to regulate bone mass. Reports have shown that decreased bone mineral density during lactation is mainly caused by perilacunar/canalicular remodeling (Qing et al., 2012; Wysolmerski, 2013). Mazur et al. have demonstrated that suppressed perilacunar/canalicular remodeling is responsible for osteoarthritis (OA). Osteocytes inside the subchondral bone show a decreased length of the canaliculus at the end stage of osteoarthritis (Mazur et al., 2019). It has been found that YAP/TAZ deletion reduces bone mass and disturbs matrix collagen content and organization by suppressing osteocyte perilacunar/canalicular remodeling (Kegelman et al., 2020). In addition, systemic inhibition of TGFβ signaling induces poor bone quality through suppression of osteocyte perilacunar/canalicular remodeling (Dole et al., 2020). What’s more, Eimear B. Dolan et al. found that thermal elevations (47°C for 1 min) can cause osteocytes apoptosis and secretory function changing (Dolan et al., 2015). As a result, it is worth investigating whether there is a correlation between temperature and the canalicular system.

Enough osteocytes are important for maintaining the bone mass. Osteocyte apoptosis caused by aging (Almeida et al., 2007; Nicks et al., 2012), alterations in the mechanical environment (Bakker et al., 2004; Aguirre et al., 2006), fatigue-induced bone microdamage (Kennedy et al., 2012), or glucocorticoid levels (Heimann and Freiberger, 1960) can decrease bone mineral density. Furthermore, the fluctuating levels of RANKL, OPG, and VEGF play a significant role in this process. With the decrease in estrogen, bone loss is caused by osteocyte apoptosis-related osteoclast activities (Emerton et al., 2010). Osteocyte apoptosis induces a decline in OPG production and promotes the release of RANKL by adjacent osteocytes. As a result, bone turnover is accelerated, leading to a lower bone density (Jilka et al., 2013). Matrix metallopeptidase 13 (MMP13) is another important factor that affects bone mass. MMP13 is known to cleave collagen I in the extracellular matrix and potentially plays a role in the turnover of articular cartilage. Evidence suggests that MMP13 is related to bone quality and plays a significant role in osteocyte perilacunar remodeling (Mazur et al., 2019). As a transmembrane glycoprotein, E11/podoplanin is vital for osteocyte differentiation, especially for the elongation of dendrites (Zhang et al., 2006). Deletion of E11 will impair the mechanical response of osteocytes and cause changes in bone mass (Staines et al., 2019; Qin et al., 2020). Accordingly, in this study, we evaluated the effects of cold-induced stress on bone mass and elucidated the potential underlying mechanisms.

## Materials and Methods

### *In vivo* Studies

Two-month-old C57BL/6J male mice were purchased from Shanghai SLAC Laboratory Animal Co. (Shanghai, China), and the study was approved by the Animal Ethics Committee of Shanghai Ninth People’s Hospital. Mice were fed commercial food and water under specific-pathogen free (SPF) conditions. For the *in vivo* study, 60 mice were haphazardly divided into two groups such as cold stimulation (cold) and room temperature (normal) groups (5 per cage); with 30 mice per group. In a nutshell, the mice in the cold group were grown at an incubator temperature of 4°C, while the normal group was nursed in the same incubators at room temperature (23°C) (Lim et al., 2012). At the end of each time point, mice were weighted and euthanasia with chloral hydrate, and then femurs and tibias were collected.

### Micro-computed Tomography Scanning

After breeding under indicated conditions and for different periods, the femurs of the mice were fixed with 4% paraformaldehyde. Samples were scanned using micro-CT (μCT 80; Scanco, Zurich, Switzerland), as described previously (Zhou et al., 2019). The cancellous bone was selected at a distance of 1.9 mm from the femoral condyle and 100 layers from the distal end of the growth plate. The micro-CT parameters were as follows: voltage of 70 kV, electric current of 114 μA, and resolution of 10 μm per pixel. The three-dimensional structural parameters studied included bone volume fraction (BV/TV), trabecular number (Tb.N), trabecular thickness (Tb.Th), and trabecular separation (Tb.Sp).

### Ploton Silver Staining

After decalcification in 10% EDTA for 3 weeks, all samples were embedded in paraffin. Sagittal sections of the medial compartment of the knee joint were cut at a thickness of 4 μm, followed by Ploton silver staining. Sections were de-paraffinized and incubated in two steps: 50% silver nitrate and 1% formic acid in 2% gelatin solution for 55 min, as previously described. The stained slides were then washed in 5% sodium thiosulfate for 10 min, dehydrated, cleared, and mounted. Consistent cortical regions were selected for evaluation in the medial and lateral areas of each specimen. Images were acquired at 100x magnification for the analysis (OLYMPUS, I×71). Quantification of the lacuno-canalicular area and canalicular length was quantified using ImageJ (Mazur et al., 2019).

### Immunohistochemical Staining

All samples were decalcified in 10% ethylenediaminetetraacetic acid (EDTA) for 3 weeks and embedded in paraffin. For microstructure observation, 4-µm-thick sagittal sections of the medial compartment of the knee joint were cut, and immunohistochemical staining with MMP13 antibody (18165-1-AP, Proteintech, Wuhan, China, 1:50), caspase-3 (9,661, Cell Signaling Technology, Inc., Danvers, MA, United States, 1:1,000) antibody, RANKL (AF462, R and D Systems, Minneapolis, MN, United States, 10 μg/ml) antibody, TRAP (ab191406, Abcam, 1:100) and Osteocalcin (ab93876, Abcam, 1:100) antibody were performed. All the antibodies are diluted by 10% goat serum.

### *In vitro* Differentiation of BAT

Brown adipocytes were isolated and cultured according to the above method (Ingram et al., 2017). Briefly, after euthanasia, the brown adipose tissue in the interscapular of 4-week-old C57BL/6 mice was collected, minced, and then digested with collagenase buffer (DMEM, 1 mg/ml collagenase I, 1% fetal bovine Serum). The preadipocytes were filtered through a 70 µm membrane and centrifuged, and then the preadipocytes were cultured with 10 ng/ml bFGF (Pepro Tech), 10% fetal bovine serum (Gibco) and pen/strep (Life Technologies) in DMEM to a confluence of 80–90%. The cells were passaged every 3 days. To get brown adipocytes, the cells were cultured with 10% fetal bovine serum (Gibco), 10 μg/ml insulin (Macklin), 1 µM dexamethasone (Sigma), 0.5 mM 3-isobutyl-1-methylxanthin, phosphodiesterase inhibitor (IBMX, Sigma), 5 µM rosiglitazone (Sigma), 1 nM T3 (Sigma) DMEM for 6 days, until brown adipocytes were formed.

### Preparation of BAT CM

In order to obtain brown adipocytes conditioned medium (BAT CM), brown adipocytes were cultured in DMEM containing 10% exosome free FBS and collected after 48 h. Centrifuge at 3 × 10^2^ g for 10 min to remove cells, and then centrifuge at 2 × 10^3^ g for 10 min and 1 × 10^4^ g for 30 min to remove cell debris and large vesicles (Cianciaruso et al., 2019; Song et al., 2019). The conditioned medium was filtered by 0.22 μm and used for the cultivation of MLO-Y4.

### Immunofluorescence

Osteocyte‐like MLO‐Y4 cells were used for studying osteocyte *in vitro*, which were kindly provided by Dr Lynda Bonewald (University of Missouri‐Kansas City, Kansas City, MO). MLO-Y4 cells were cultured with α‐minimum essential medium (α‐MEM; Hyclone) containing 5% fetal bovine serum (FBS; Gibco), 5% calf bovine serum (CBS; Gibco), and 1% penicillin/streptomycin (Sigma‐Aldrich, St. Louis, MO) on rat tail collagen type I-coated (Solar bio, Beijing, China) dish. The experiment design included two groups: control group (Ctrl) and conditional medium group (CM). MLO-Y4 cells (1×10^5^) were seeded on 3.5 cm dishes coated with type I rat tail collagen (Corning). The medium was changed when the cell density reached 50%. Control groups continued to be cultured in the growth medium, while CM groups were cultured with brown adipocyte conditional medium and growth medium 1:1. After 24 h, the cells were fixed with 4% paraformaldehyde (PFA) for 30 min, washed with PBS and then gradient incubated in 0.03% Triton X‐100 and blocking buffer (1× TBST, 10% normal goat serum) for 1 h at room temperature (23 °C). E11 antibody (Abcam, ab11936, 1:200) was added to the wells and overnight at 4°C. After incubation with secondary antibody for 1 h, TRITC-phalloidin (Kingmorn, China) and DAPI (Thermo Scientific, US) were used for cytoskeleton and nuclear staining, then cells were observed and captured with confocal fluorescence microscopy.

### Quantitative reverse‐transcription Polymerase Chain Reaction (qRT-PCR)

Total RNA was extracted with TRIZOL reagent (Thermo Scientific, United States). After measuring the concentration, Quant script RT Kit (Promega, Madison, WI, United States) was used to convert total RNA into complementary DNA. A 10 ul PCR reaction system composed of cDNA and SYBR Premix Ex Taq Mix (Selleck) was used to detect the expression level of messenger RNA (mRNA) in the Real-Time PCR system (Light Cycler 2.0; Roche Diagnostics GmbH, Mannheim, Germany). The primer sequences are shown in Table 1.

**TABLE 1:** Primer sequences for the quantitative reverse‐transcription polymerase chain reaction.

Target genes	Forward (5′-3′)	Reverse (5′-3′)
Gapdh	AGG​TCG​GTG​TGA​ACG​GAT​TTG	TGT​AGA​CCA​TGT​AGT​TGA​GGT​CA
E11	ACC​GTG​CCA​GTG​TTG​TTC​TG	AGC​ACC​TGT​GGT​TGT​TAT​TTT​GT
Ocn	CTG​ACC​TCA​CAG​ATC​CCA​AGC	TGG​TCT​GAT​AGC​TCG​TCA​CAA​G
Sost	AGC​CTT​CAG​GAA​TGA​TGC​CAC	CTT​TGG​CGT​CAT​AGG​GAT​GGT
Runx2	CCG​GGA​ATG​ATG​AGA​ACT​A	ACCGTCCACTGTCACTTT

### Statistical Analysis

Statistical analyses of data were performed using GraphPad Prism 5.0 (GraphPad Software Inc., San Diego, CA, United States). Each group have at least five mice. All quantitative values are presented as the mean ± standard deviation, and differences were evaluated by *t*-test or two-way ANOVA, followed by Bonferroni correction for multiple comparisons. Statistical significance was set at *p* < 0.05.

## Results

### Exposure to Cold Temperature Results in Bone Mass Variations

**FIGURE 1:**
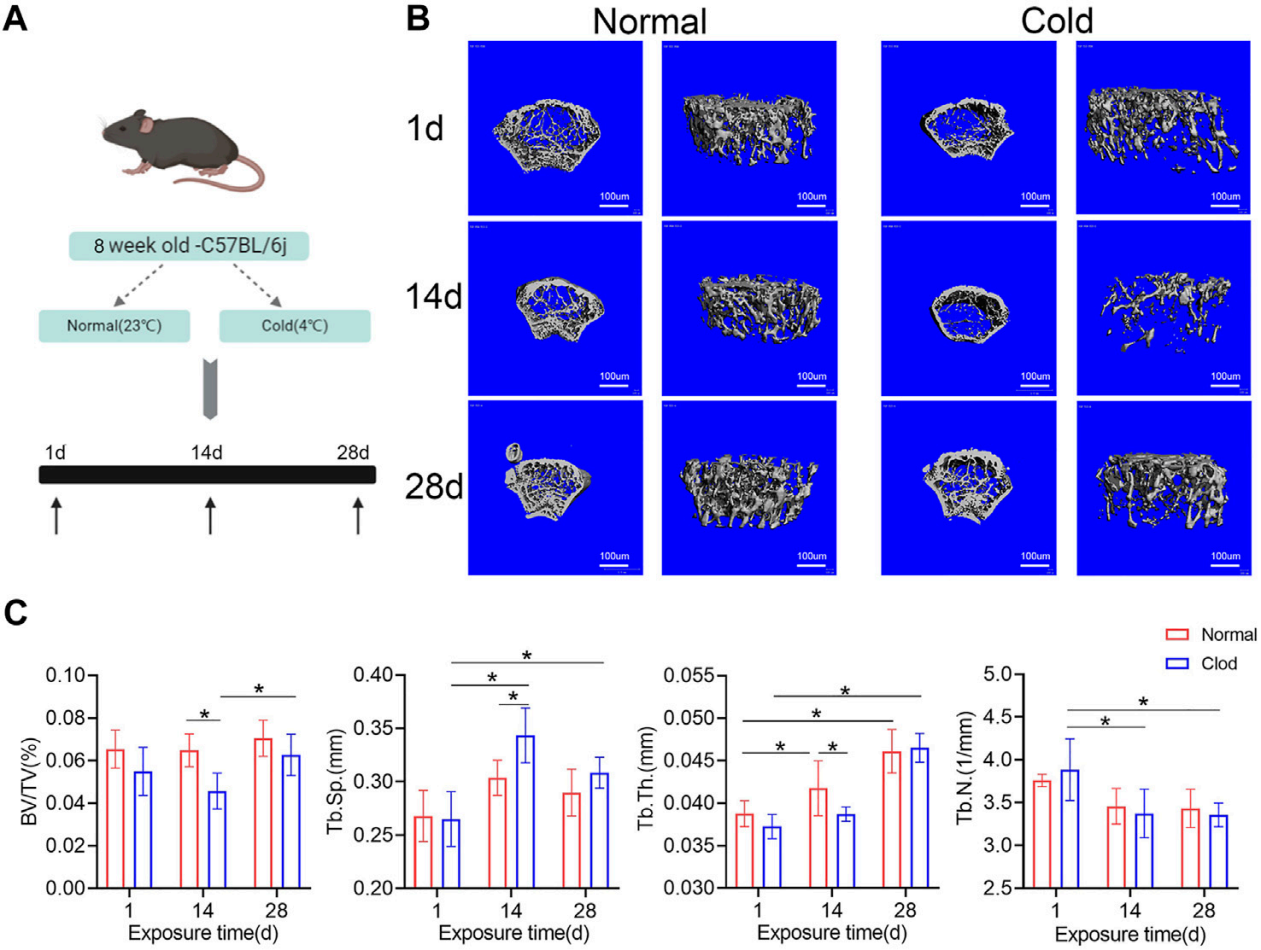
**I**nfluence of cold-stress on bone mass in a time-dependent manner. **(A)** Schematic flow of the experimental design. Mice were nursed at different temperatures (4 and 23°C) and sacrificed after 1, 14 or 28 days. **(B)** The representative micro-CT 3D reconstruction images of different groups. **(C)** Trabecular bone microarchitecture of femurs showing bone volume/total volume (BV/TV), trabecular number (Tb.N), trabecular separation (Tb.Sp), and trabecular thickness (Tb.Th). Data are shown as mean ± SD (n = 5 per group). Significance (*p* value) is calculated using two-way ANOVA, *p < 0.05; Abbreviations: 1 day (1d), 14 days (14d), 28 days (28d), Normal: mice in the room temperature, Cold: mice in the 4°C.

To determine the effects of cold-induced stress on bone mass, the C57BL/6J male mice were exposed to cold (4°C) or room (23°C) temperature for different time intervals (Figure 1A). Their bone mass parameters were calculated using micro-CT (Figure 1B). As shown in Figure 1C, the bone volume fraction (BV/TV) of the mice belonging to the cold-stress group reduced in 14 days compared to that of mice of the room temperature/control groups; BV/TV same recovered after 28 days. Further, the trabecular thickness (Tb.Th) was found to be lower at 14 days in the mice belonging to the cold-stress group than in the control group, which is consistent with the increased trabecular separation (Tb.Sp). The mice were weighted at different time points. As shown in Figure 1D, there was no significant change in the body weight of the mice over time. These results indicate that stimulation with cold temperature has a negative effect on the bone mass after short-term cold exposure (14 days), but with prolonged exposure, this effect gets debilitated. These findings suggest the existence of an adaptation process in bones that gets activated upon exposure to cold temperatures.

### Osteocyte Lacuna-Canalicular System Exhibits Variation During Exposure to Cold Temperature

**FIGURE 2:**
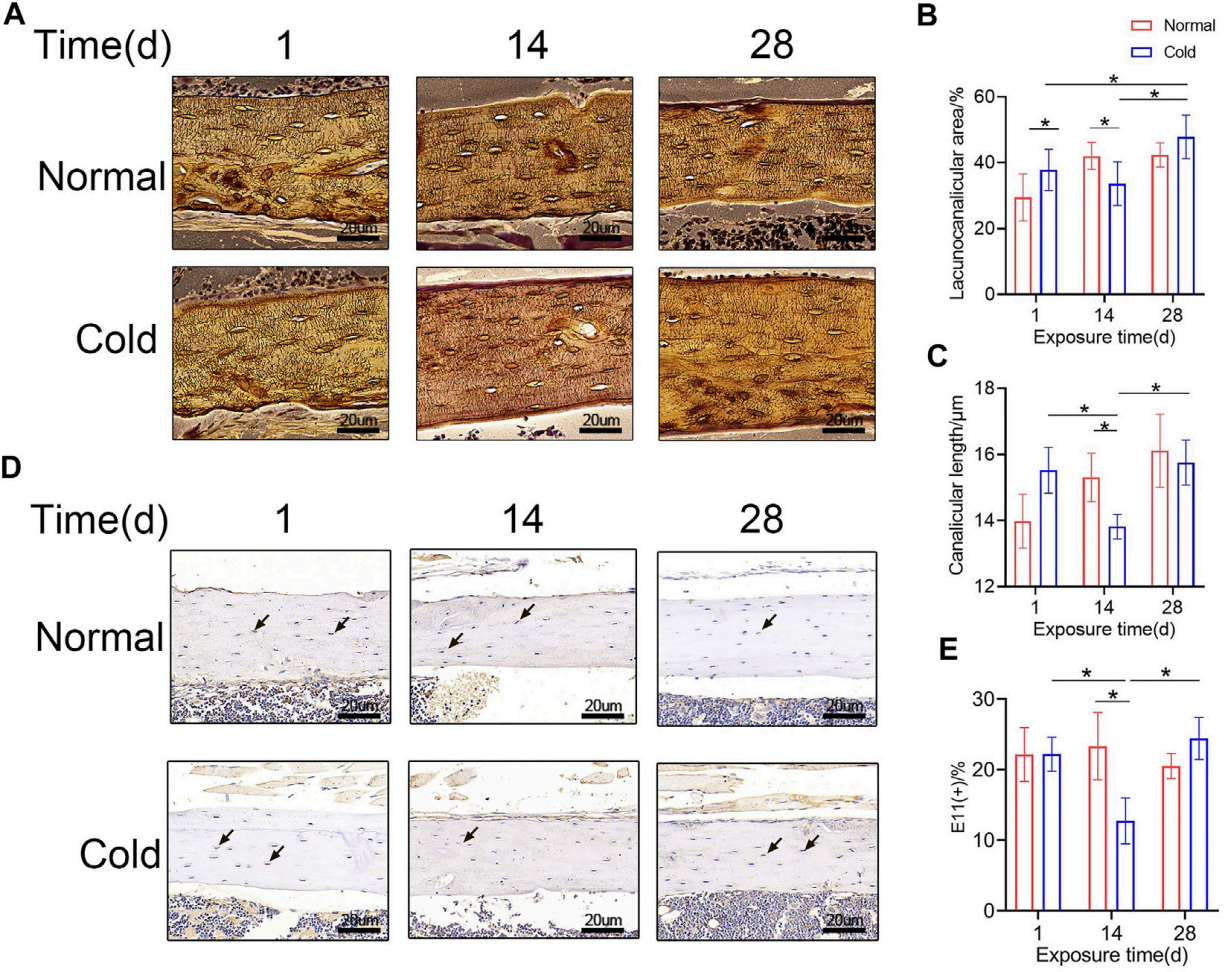
Osteocyte canaliculus shows alterations with exposure to cold temperature. **(A)** Ploton silver stain was performed to show the canalicular network in cortical bone. **(B–C)** Lacuno-canalicular area and canalicular length were measured and the differences among the groups are shown. **(D)** Immunohistochemical stain of E11 in the femurs was performed. **(E)** E11-positive osteocytes are calculated and statistic results are shown. Black arrows: E11 positive osteocytes. Data are shown as mean ± SD (*n* = 5 per group). Significance (*p* value) is calculated using two-way ANOVA, *p < 0.05.

To study the effect of cold-induced stress on the lacuna-canalicular system, the C57BL/6J mice were exposed to cold (4°C) or room (23°C) temperature for different time intervals and the bone lacunar-canalicular system was studied using the ploton silver staining technique as shown in Figure 2A. The lacuno-canalicular length and area were counted and are shown in Figures 2B,C. The results revealed that after 14 days of cold stimulation, canalicular area and length in the femur were reduced compared to that in control group mice. Further, the lacuno-canalicular area was enlarged and the canalicular length was recovered after 28 days of exposure (Figures 2B,C). E11, which was also known as podoplanin, is a cell membrane protein that can expressed in osteocytes (Nose et al., 1990; Schacht et al., 2003). As shown in Figure 2D,E, E11-positive osteocytes number reduced in the 14 days of the cold treated group, which consisted with the results of ploton silver staining. The lacuno-canalicular system is an important component of osteocytes and is a mechanical sensor that indirectly affects bone mass. The changes that occur during the cold-exposure treatment suggest that the alteration in bone mass may be caused by the variation in mechanical response ability. Decreased canalicular length at 14 days was consistent with the lower bone mass in the cold group unlike in the control group mice. Furthermore, prolonged exposure to cold results in the recovery of bone mass, with elongation and enlargement of the lacuna-canalicular area.

### Cold Exposure Causes Osteocytes Apoptosis

**FIGURE 3:**
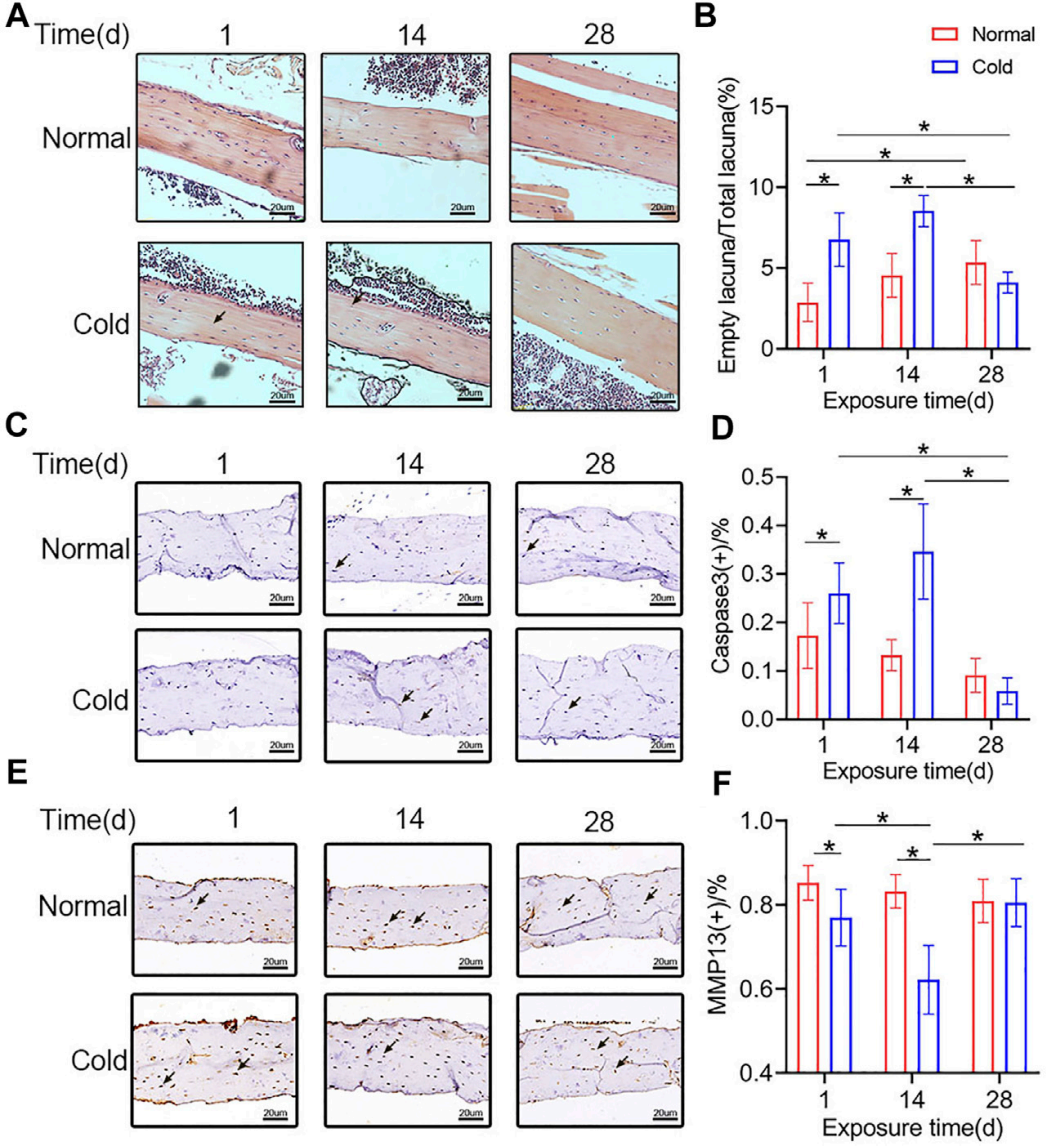
Cold exposure induces osteocytes apoptosis. **(A)** Representative pictures of H and E staining in the femurs. **(B)** Quantification of empty lacunae in the **Panel A**. **(C)** Immunohistochemical stain of Caspase3 in the femurs was performed. **(D)** Caspase3-positive osteocytes are calculated and statistically represented. Black arrows: Empty lacuna or Caspase3 positive osteocytes. **(E)** Immunohistochemical stain of MMP13 in the femurs was performed. **(F)** MMP13-positive osteocytes are calculated and statistic results are shown. Black arrows: MMP13 positive osteocytes. Data are shown as mean ± SD (*n* = 5 per group). Significance (*p* value) is calculated using two-way ANOVA, *p < 0.05.

To study the effect of cold-stress on the fate of osteocytes in cold- and room temperature-treated mice, H and E staining and immunohistochemical studies were performed. The H and E staining was employed to visualize the empty bone lacuna (Figure 3A). The results revealed that in the cold group, the proportion of empty lacunae increased on days 1 and 14 (Figure 3B). The immunohistochemical analysis was performed to determine the expression level of Caspase3 (Figure 3C) *in vivo*. As the expression of Caspase3 is positively correlated with apoptosis, Caspase3-positive osteocytes were considered apoptotic. Figure 3D shows that the expression level of Caspase3 in the cold-stress group was found to be augmented at 14 days compared to that in the control group, and there was no significant difference between the two groups after 28 days of exposure. Besides, immunohistochemical analysis was performed to determine the expression level of MMP13 (Figure 3E) *in vivo* in both the groups of mice. As shown in Figure 3F, MMP13-positive osteocytes numbers decreased after 1 and 14 days in the cold environment. Changes in the empty lacuna, the number of Caspase3-and MMP13 positive osteocytes caused by the different temperatures indicate that exposure to cold can influence bone mass through osteocytes.

### Cold Affects the Secretory Function of Osteocytes

**FIGURE 4:**
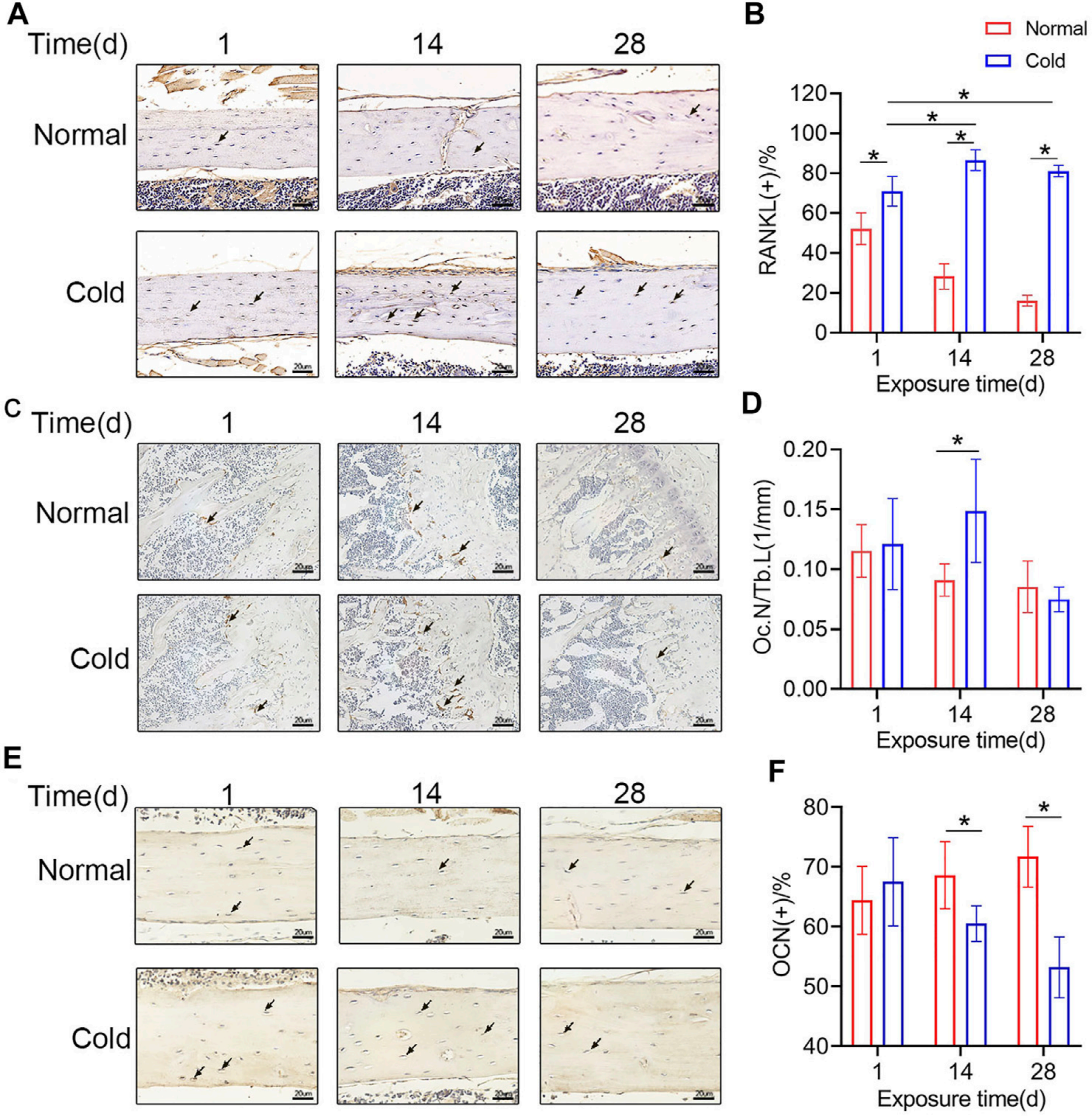
Cold affects the secretory function of osteocytes **(A)** Immunohistochemical staining for RANKL in the femurs was performed. **(B)** RANKL-positive osteocytes were calculated and are statistically represented. **(C–D)** Immunohistochemical staining for TRAP in the femurs was performed and Oc. N/Tb.L were calculated. **(E–F)** Immunohistochemical staining for OCN in the femurs was performed and OCN positive osteocytes were calculated. Black arrows: RANKL-, TRAP-, or OCN-positive osteocytes. Data are shown as the mean ± SD (*n* = 5). Significance (*p* value) was calculated using two-way ANOVA, *p < 0.05.

As mentioned previously, cold exposure induced osteocyte apoptosis at 14 days. Reports show that dead osteocytes exhibit higher RANKL expression in the neighboring cells. To explore if there was a change in the level of RANKL, immunohistochemical staining was performed in the mice belonging to the cold/control groups, and representative pictures are shown in Figure 4A. Unsurprisingly, the proportion of RANKL-positive osteocytes was increased in the cold-stimulated group (Figure 4B), which is consistent with the variation trend of apoptotic osteocytes. Immunohistochemical staining were performed to show TRAP and osteocalcin (OCN) positive cells in bone tissue. As shown in Figure 4C and Figure 4D, TRAP positive osteoclasts and Oc. N/Tb.L were calculated, the results indicated that Oc. N/Tb.L were increased after 14 days in cold environment, which may explain the bone loss in the same time point. What’s more, OCN positive osteocytes were decreased with the stimulation of cold exposure in the 14 and 28 days (Figures 4E,F). All of this makes it even more convincing that temperature plays a role in bone remodeling, and that’s probably initiated by its effect on osteocytes.

### BAT CM Increases the Length of Osteocyte‘s Dendrites

**FIGURE 5:**
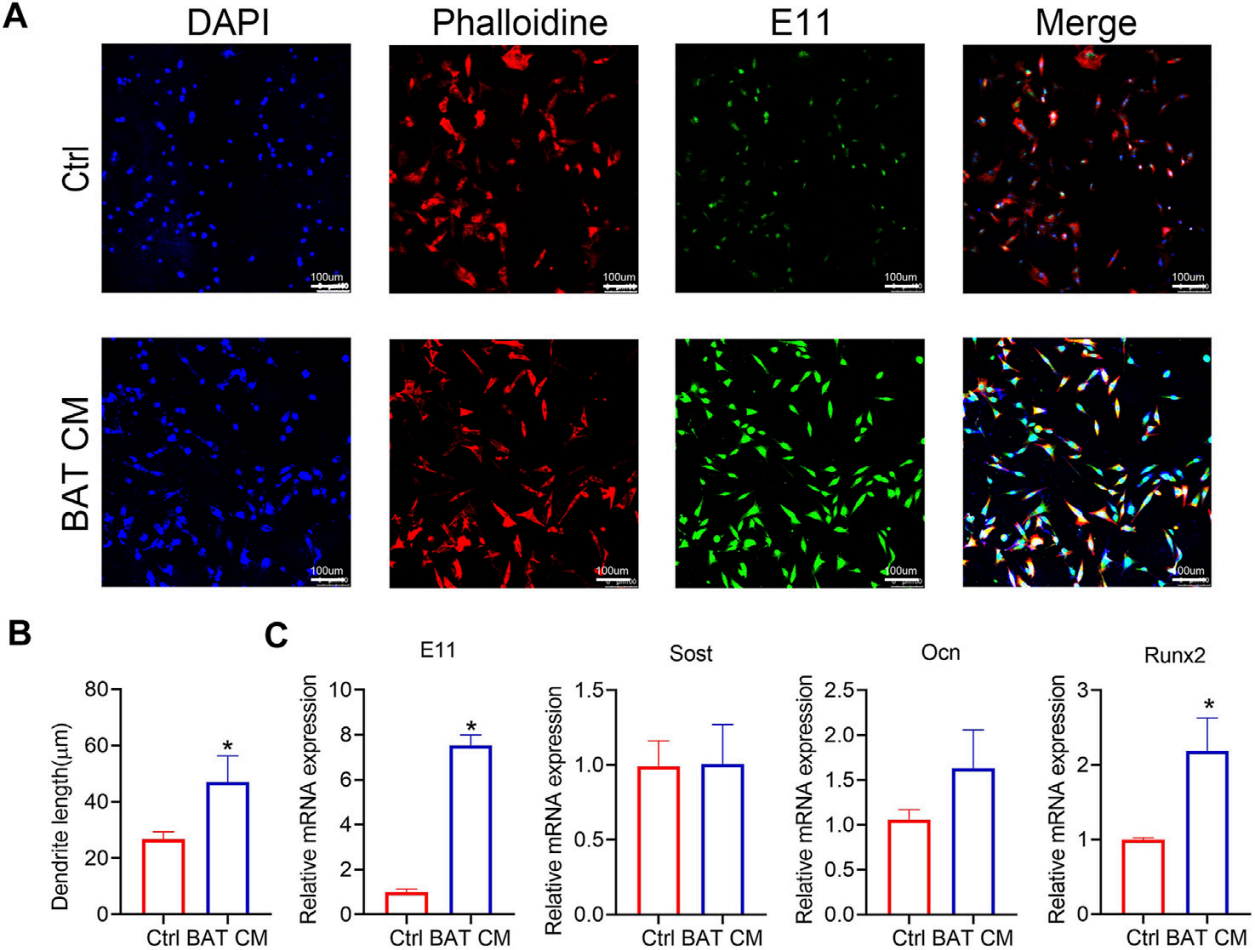
BAT CM increase the length of osteocyte‘sdendrites. **(A,B)** Immunofluorescent staining was performed to show the morphology of MLO-Y4 and dendrites length are calculated. **(C)** Relative mRNA expression levels of E11, Sost, and Ocn were detected by RT-PCR. Data are shown as mean ± SD (n ≥ 3 per group). Significance (*p* value) is calculated using student’s t-test, *p < 0.05; Abbreviations: 1 day(1d), 14 days (14d), 28 days (28d), Normal: mice in the room temperature (23°C), Cold: mice in the 4°C.

BAT CM was added to the culture medium of MLO-Y4, and immunofluorescent staining was performed to show the morphology of MLO-Y4 (Figure 5A). As shown in Figure 5B, dendrites of MLO-Y4 in the BAT CM group were longer than Ctrl group. Besides, relative mRNA expression levels of E11, Sost, and Ocn were promoted by BAT CM(Figure 5C).

## Discussion

After exposure to the cold environment, the C57BL/6J mice showed a reduction in bone mass at 14 days as compared to the control conditions, but the changes were recovered at 28 days. Further studies demonstrated that there was an increased apoptotic osteocyte, shorter canalicular length, increased osteoclasts and changed RANKL and OCN expression level at 14 and 28 days in the cold group, which may be the important factors inducing undulation of bone mineral density.

The relationship between temperature and bone mass has fascinated scientists in the past few decades. It has been reported that nursing at 22°C can induce bone loss in C57BL/6J and C3H/HeJ mice (Iwaniec et al., 2016; Robbins et al., 2018). Environmental factors may play a significant role in the energy metabolism of laboratory animals (Bektas et al., 2018). The reasons that have been considered are the reduced blood flow in the hind limb and changed volume of brown adipose tissue. Serrat et al. have attributed the shorter hind limb in a cold environment to cell proliferation and matrix production in cartilage (Serrat et al., 2008). Animals in warmer places have longer limbs and stable bone mineral density (Serrat et al., 2008; Chevalier et al., 2020). In our study, when 2-month-old male C57BL/6J mice were exposed to 4°C for 14 days, bone loss was observed. However, when cold stimulation was prolonged to 28 days, the bone mass was partly recovered. Consistently, there have been reports that mice housed at 22°C for 9 weeks show lower BV/TV than thermoneutrality. Although 22°C is much higher than 4°C, both are relatively low temperatures compared with room temperature. As bone mass showed no fluctuation in Robbins’s study, which may be attributed by the difference of observation interval (Robbins et al., 2018).

To illustrate the potential mechanism, osteocyte canalicular networks were stained with ploton silver, and the statistical results showed shorter canalicular length in the cold group after 14 days of cold exposure as compared to control temperature. Immunohistochemical analysis was performed to test the E11 and MMP13 expression *in vivo*, and the results showed that short-term cold exposure was negatively related to E11 and MMP13 expression. As E11 was mainly expressed in the cell membrane of osteocytes, which has a positive effect on bone mass (Nose et al., 1990; Zhang et al., 2006), it is reasonable to speculate that cold can cause bone loss by affecting the expression of E11. Further, with the enlargement of brown adipose, restored E11 expression levels lead to recovery of bone mass (Wetterwald et al., 1996; Hadjiargyrou et al., 2001; Jung et al., 2019). Osteocytes can regulate bone mass through their lacuno-canalicular networks. They can secrete numerous factors, such as cathepsin K (CatK), prostaglandin, and matrix metalloproteinases (MMPs), and participate in perilacunar/canalicular remodeling processes (Bonewald, 2011; Qing et al., 2012; Tang et al., 2012; Mazur et al., 2019). Prior studies have noted the importance of perilacunar/canalicular remodeling (PLR) in osteoarthritis (Mazur et al., 2019). As mentioned above, the lacuno-canalicular network is the mechanical sensory component of the bone. Changes in the length and area disturb the metabolic balance of bone. During the lactation period, osteocytes have shorter dendrites and reduced bone mineral density (Wysolmerski, 2013). Mice in the 4°C environment had shorter canaliculus and smaller canalicular space as compared to the control conditions, which may partially explain the lower bone mass at 14 days.

H and E staining showed an increased number of empty lacunae after cold stimulation, which may be caused by osteocyte apoptosis. To determine whether cold-stress can influence osteocyte viability, Caspase-3 positive cells in the cortical bone were counted and it was found that the number of apoptotic osteocytes in the cold group was higher than that in the normal group. As the initiator of bone remodeling, the osteocyte number is positively related to bone mass and apoptosis of which results in bone loss. The relationship between apoptotic osteocytes and bone mass has been well studied; osteoclastogenic cytokines released by apoptotic osteocytes activate osteoclasts and lead to bone resorption (Jilka et al., 2013). Aging, hormones, glucocorticoids, and mechanical stimulation have been found to play a critical role in osteocyte apoptosis (Bakker et al., 2004; Almeida et al., 2007; Jilka et al., 2013; Silva et al., 2020). Therefore, it is reasonable to assume that extremely low temperatures initiate the apoptosis of osteocytes and active osteoclasts. As one of the major cytokines secreted by osteocytes (Nakashima et al., 2011), RANKL is significantly increased in the cold as compared to the control group. RANKL can be elevated in many ways. Increased TNF-α and IL-6 expression levels in the circulatory system promote the formation of RANKL, which directly activates osteoclasts (Wu et al., 2017; Marahleh et al., 2019). Additionally, mechanical loading regulates the expression of RANKL by promoting the release of PGE2 (Iolascon et al., 2013; Uda et al., 2017). Apoptotic osteocytes facilitate the production of RANKL by neighboring cells (Hughes et al., 2020). In our study, RANKL was elevated in the cold group, which partly explains the decrease in bone mineral density. Research have shown that bone remodeling can be affected by temperature (Iwaniec et al., 2016; Ziętak et al., 2016), which may cause changed osteoclast number and osteoblast activity. Osteoclasts, the TRAP positive cell type and mainly causing bone resorption, were increased by cold stimulation in the 14 days. As a vitamin K-dependent protein, OCN seems to promote the osteoblast-to-osteocyte transition and also limit osteoclastogenesis (Atkins et al., 2009; Palermo et al., 2017). With the lengthening of cold stimulation time, OCN positive osteocytes were decreased in the 14 and 28 days. All of this may provide reasons for the decreased bone mass in the cold stimulation group.

In contrast to cold stimulation, warmth exposure has a positive effect on bone development and bone mass (Romsos et al., 1985; Serrat et al., 2008). Intestinal flora inter-communicates with host physiology, and environmental variation influences microbial composition, while the fluctuation of the microbiota in the gut induces changes in organ metabolism (Chevalier et al., 2015; Ziętak et al., 2016). Reports have shown that intestinal flora is an important regulator of bone metabolism (Jones et al., 2018; Li et al., 2019). Chevalier et al. have shown that transplantation of warm microbiota can protect ovariectomized mice from bone loss (Chevalier et al., 2020). It is reasonable to speculate that some changes occur in the internal microorganisms in cold-treaded mice. In addition, the gut-brain-bone axis may play a role in this process (Quach and Britton, 2017).

The sympathetic nerve is considered to be an effector of bone remodeling. The sympathetic nerve is activated in a cold environment (Nedergaard and Cannon, 2014), which can decrease bone mass by regulating the balance between osteoblasts and osteoclasts (Bonnet et al., 2007; Fonseca et al., 2014; Vignaux et al., 2015). In addition, brown adipose volume is positively related to cold stimulation; and nursing mice at 4°C may cause increased UCP1 expression and enlarged brown adipose tissue (Cannon and Nedergaard, 2004; Bartness et al., 2010; Lim et al., 2012). Hence, the energy consumed by adipose tissue has been reported to be positively related to bone (Devlin, 2015; Lidell and Enerbäck, 2015). This might be an important factor in maintaining bone mineral density. Similarly, we confirmed BAT CM had a positive influence on the dendrites of osteocytes in this study. Qing et al. have reported that IL-6, which is mainly secreted by brown adipose tissue, is one of the most important factors under stress (Qing et al., 2020). Thus, there may be high levels of IL-6 that led to the activation of osteoclasts in the bone loss period (Jilka et al., 1992; Lazzaro et al., 2018), but with the augmentation of brown adipose, the bone mass gets reduced.

There still have some limitations in this study. First, we attributed the fluctuation of bone mass in cold treated group to the changed osteocytes apoptosis and lacuno-canalicular area, which were not confirmed *in vivo* and vitro. Besides, the waved bone mass in the cold stimulated group can’t be explained very well, and the reason behind it should be explored. What’s more, we just use mice to detect the relationship between bone mass and temperature, there may have some difference in human.

In this study, E11, MMP13, Caspase3, RANKL TRAP and OCN expression levels change with prolonged cold exposure, which may contribute to the bone mass change during cold exposure. Low temperatures exposure induced brown adipose accumulation can influence bone mass through affecting osteocytes.

## Data Availability

The raw data supporting the conclusions of this article will be made available by the authors, without undue reservation.
